# The application of a hollow trephine in femoral retrograde intramedullary nailing technique

**DOI:** 10.1186/s12891-023-06351-8

**Published:** 2023-03-30

**Authors:** Xiang Yao, Hongyuan Liu, Minjie Hu, Chong Wang, Dejun Liu, Jianping Hu, Jilei Tang

**Affiliations:** 1 Department of Orthopaedics, The Affiliated People’s Hospital of Jiangsu University, Zhenjiang, 212000 Jiangsu China; 2 Jiangsu University, Zhenjiang, 212000 Jiangsu China; 3 Department of Orthopaedics, Tengzhou Cengde Department of Orthopedics Hospital, Zaozhuang, 277000 Shandong China; 4 Department of Orthopaedics, Qidong Hospital of Traditional Chinese Medicine, Nantong, 226200 Jiangsu China

**Keywords:** Mid-distal femur fractures, The hollow trephine, Retrograde intramedullary nailing, Bone debris

## Abstract

**Purpose:**

The purpose of this study was to describe and evaluate the use of a specially designed hollow trephine to create the entry point through the femoral condyle during retrograde interlocking intramedullary nailing for femoral fractures.

**Methods:**

From June 2019 to December 2021, we treated 11 patients (5 men, 6 women; mean age, 64 years; age range 40–77 years) with mid-distal femoral fractures by retrograde intramedullary femoral nailing using a self-designed hollow trephine for femoral condyle reaming and cancellous bone harvesting. The mode of all the nails is static. Patients were followed up at 1, 4, 8, and 12 weeks and for at least 6 months after surgery. The healing process and heterotopic ossification were evaluated by imaging. Partial weight bearing was permitted during the recovery period and complete weight bearing was permitted after clinical healing of the fracture displayed by X-ray.

**Results:**

The operation was successful in all patients. Over mean follow-up of 9.3 months (range, 6.0–12.0 months), all patients achieved clinical healing within three months. There were no complications such as knee joint infection, heterotopic ossification, knee joint adhesion and wedge effect.

**Conclusion:**

The use of the hollow trephine during femoral retrograde intramedullary nailing helps avoid postoperative complications such as heterotopic ossification, knee joint adhesions, and wedge effect. It also facilitates bone graft harvesting.

## Introduction

A fracture of the mid-distal femur is a common injury. For simple femoral shaft fractures, intramedullary nail fixation is the gold standard treatment [1]. Intramedullary nailing can be by the antegrade or retrograde technique. Retrograde intramedullary nailing is suitable for mid-distal extraarticular fractures (33-A) and sometimes also for simple articular fractures (33-C1, 33-C2). In the standard operation, the orthopedic surgeon cuts the knee capsule and uses a solid or hollow bit with a guide needle to open and expand the femoral condyle. Because the entrance of the retrograde intramedullary nail is located deep within the knee joint cavity, it is difficult to remove all the debris generated by the drilling procedure. The residual bone debris is an important cause of postoperative heterotopic ossification [2] and joint stiffness [3, 4]. Heterotopic ossification in patients with floating knee injury is significantly more common after retrograde intramedullary nailing than after antegrade intramedullary nailing.

The use of a hollow trephine instead of the ordinary solid reamer will allow removal the bone ring at the entry port with less generation of bone debris. The hollow trephine has been used during proximal femoral intramedullary nailing procedures [5]. Cepni et al. have also tried reaming with a manual trephine and closed the channel with a bone bolt after fixation [6]. We have designed a hollow trephine with limited depth measurement and used it during retrograde intramedullary femoral nailing in 11 patients. The purpose of this paper is to describe the application of the newly designed hollow trephine in retrograde intramedullary nailing of the femur.

## Materials and methods

### Patients

From June 2019 to December 2021, 11 patients with distal femoral fractures received femoral retrograde intramedullary nailing using the hollow trephine at our hospital. Imaging examinations were performed on all patients before surgery. All 11 patients had mid-lower femoral fractures. One patient also had a supracondylar femoral fracture and one had a patellar fracture. The injuries were sustained in traffic accidents (n = 5) or falls (n = 6). This study was approved by the local institutional review board, and all patients provided informed consent before surgery.

### Design of the hollow trephine

**Fig. 1:**
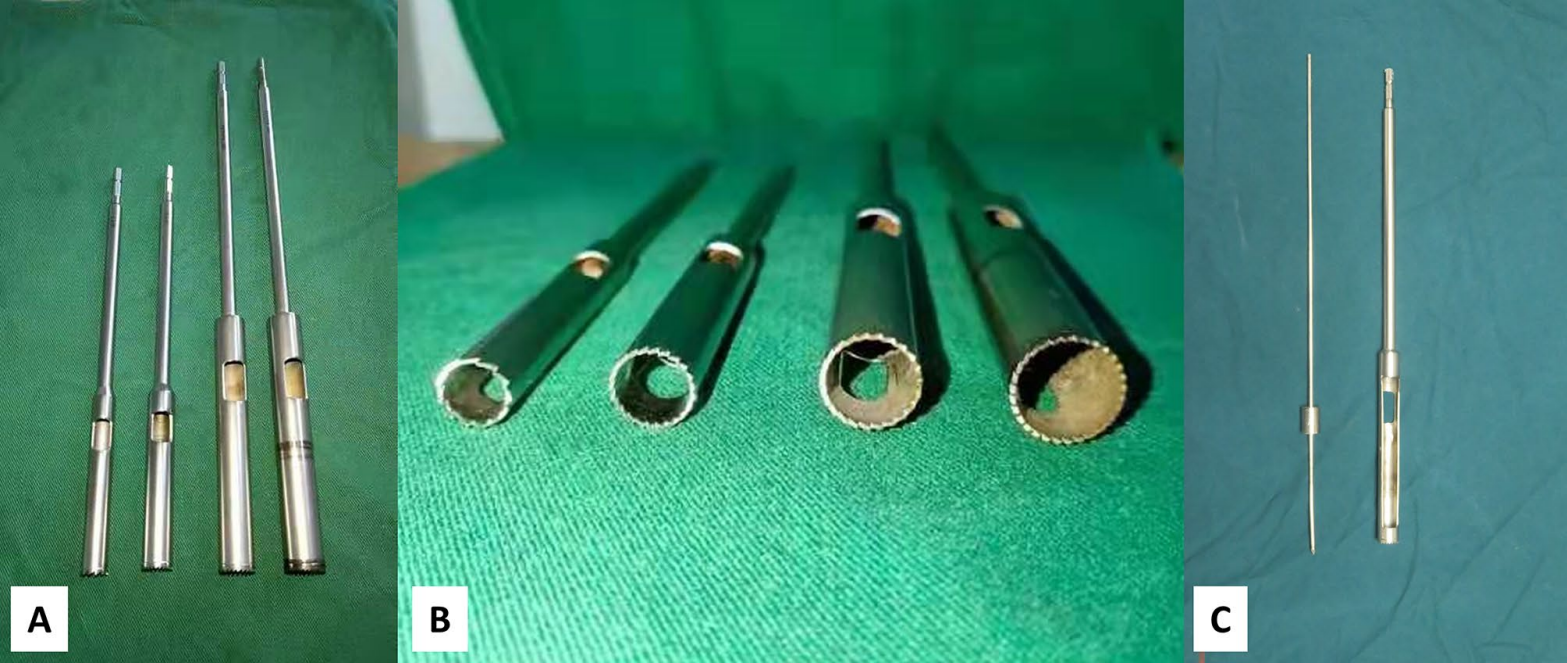
A: The hollow trephine consists of a trephine bit and a trephine rod B: The serrations and holes at the side wall of the trephine bit C: The guide pin with depth-limiting device and the trephine

The hollow trephine is made of medical-grade stainless steel and consists of two parts: a trephine bit and a trephine rod. The trephine bit has a serrated opening at the proximal end and holes on the side walls. The distal end is the trephine rod, which can be connected to the electric drill (Fig. 1).

### Surgical technique

**Fig. 2:**
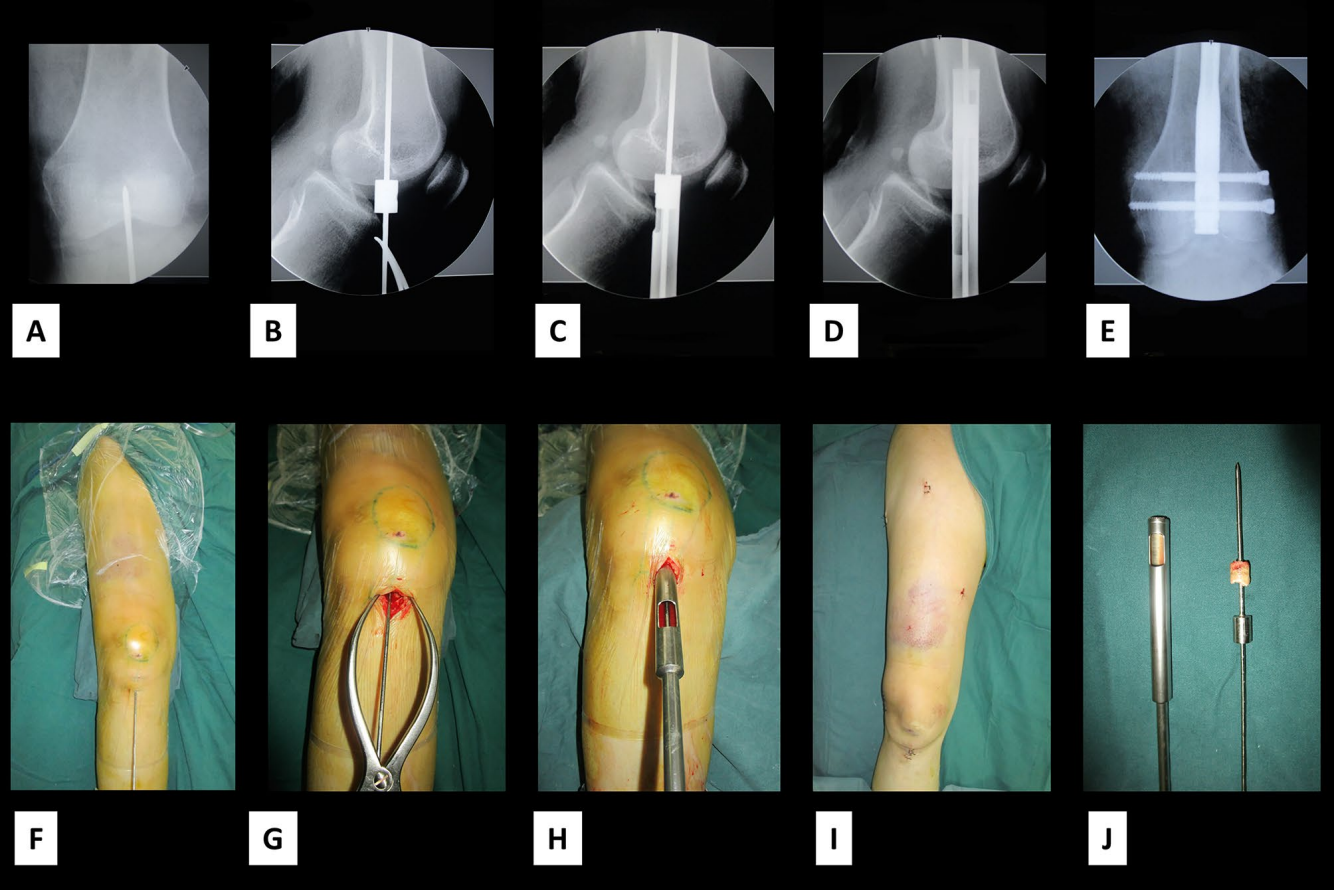
A-E: Intraoperative anteroposterior and lateral radiographs show the application of a hollow trephine F-I: The incision J: The hollow trephine and harvested bone bolt

**Fig. 3:**
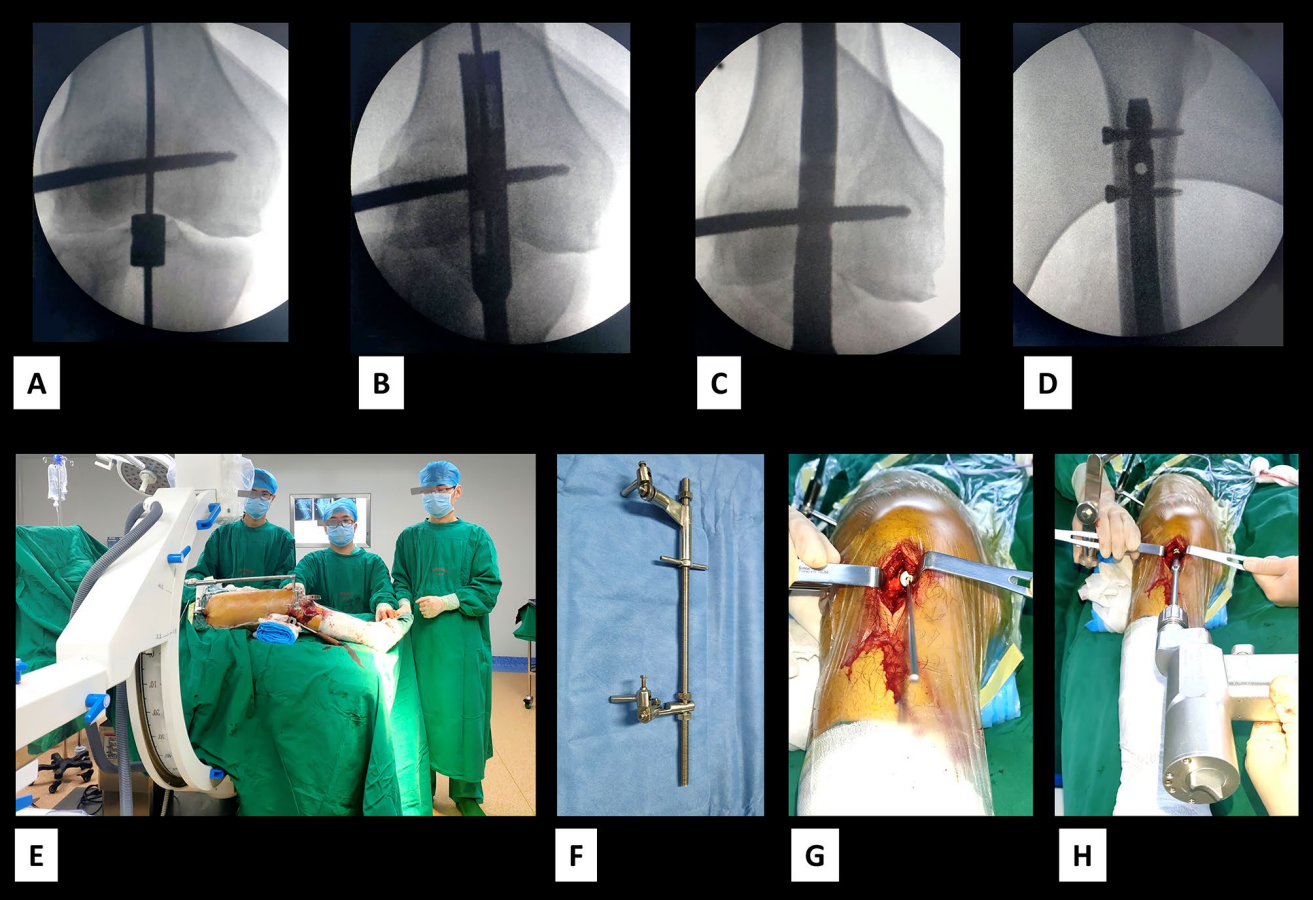
The hollow trephine can also be used in unilateral retractor-assisted surgery A-D: Anteroposterior and lateral radiographs showing intraoperative application of the hollow trephine E, F: The use of a unilateral retractor. (the images provided in Fig. 3E are authors images) G, H: Open-hole reaming of the distal femur using a hollow trephine

All operations were performed under general anesthesia. The skin was prepared and the area draped. A 3–4 cm longitudinal incision was made in the middle of the knee. If the incision is small, we incise the patellar tendon directly, and if the incision is large enough to place the retractor hook, we retract it to the side. With the knee joint flexed about 30°, the central pin with a depth-limiting device was inserted into the medullary canal. The position of the pin was checked on radiograph (Fig. 2). The guide pin was pushed forward, and the hollow trephine was connected to the drill bit to open the distal femur. Conventional reaming, closed reduction, pinning, and proximal and distal locking were performed, with fluoroscopy used to monitor pin position and satisfactory fracture reduction (Fig. 3). If fracture reduction was not satisfactory, open reduction was performed. Patients were followed up at 1, 4, 8, and 12 weeks and for at least 6 months after surgery. The healing process and heterotopic ossification were evaluated by imaging. Partial weight bearing was permitted during the recovery period and complete weight bearing was permitted after clinical healing of the fracture displayed by X-ray.

## Results

**Fig. 4:**
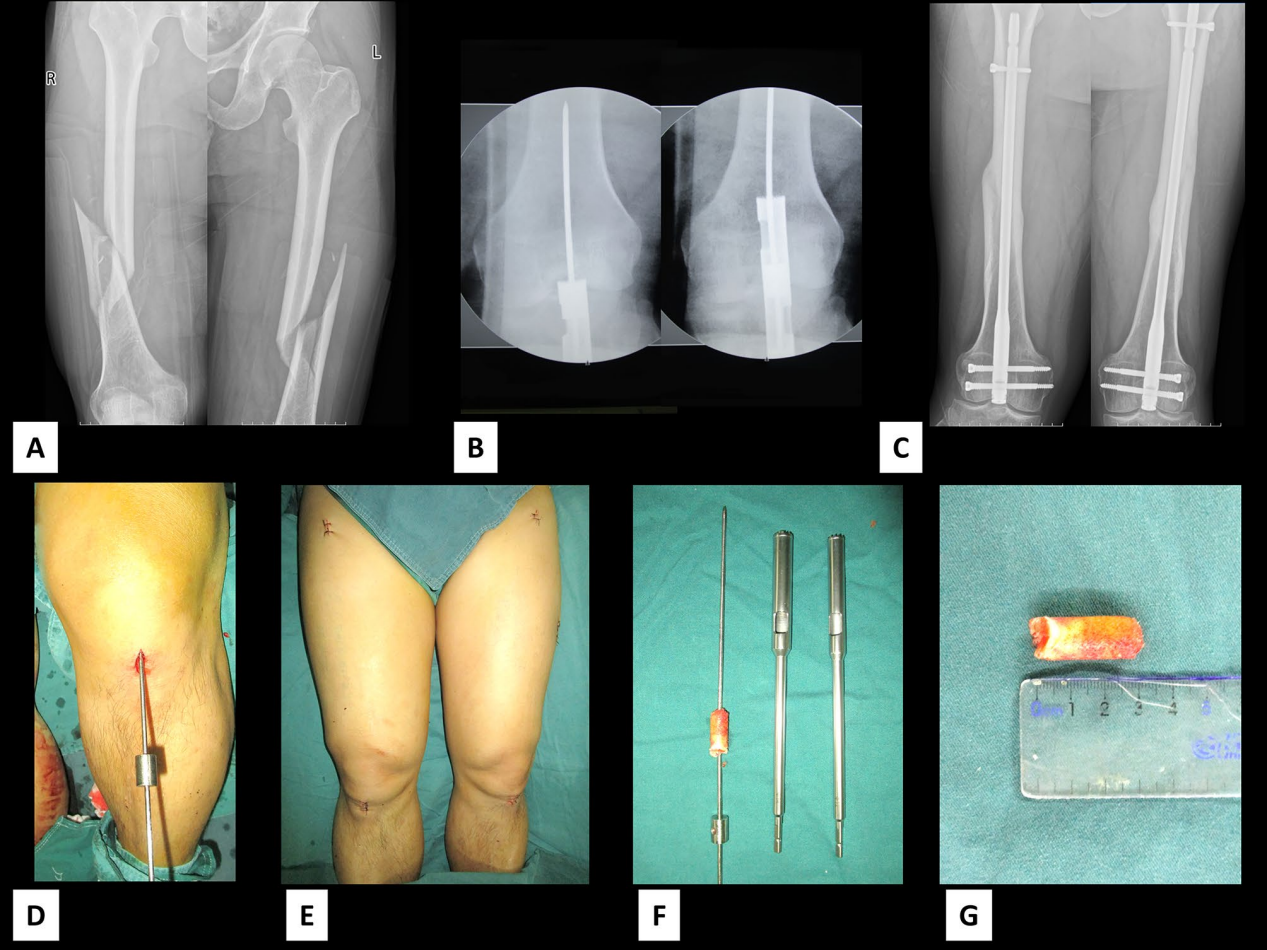
Application of a hollow trephine in a patient with bilateral femoral fractures A-C: Preoperative and postoperative anteroposterior radiographs of a patient D-G: Bone bolt obtained during surgery

A total of 11 patients (6 women, 5 men) with mid-distal femoral fractures underwent retrograde intramedullary nailing using the hollow trephine. The mean age of the patients was 64 years (age range, 40–77 years). Over mean follow-up of 9.3 months (range, 6–9 months), all patients, including one patient with bilateral femoral fractures, achieved clinical healing within three months (Fig. 4). There were no complications such as knee joint infection, stiffness, heterotopic ossification, knee joint adhesion, or wedge effect. Postoperatively, all patients achieved knee range of motion approximately 140° in flexion.

## Discussion

In this paper, we introduce the application of a hollow trephine to create the entry port during retrograde intramedullary nailing of mid-distal femoral fractures. We achieved good functional outcomes, with no complications, in all 11 patients treated by our technique. Compared with other traditional treatments, the use of a hollow trephine for surgery has many advantages. This is a practical surgical tip, which could reduce bone debris, harvest grafts and avoid the wedge effect.

Whether anterograde or retrograde intramedullary nailing is better for mid-distal femoral fractures has always been controversial. Some scholars worry that the retrograde approach interferes with the normal structure of the knee joint and leads to intra-articular adhesions and loss of knee joint function [7–12]. However, no knee adhesions or heterotopic ossification occurred in our small cohort, and functional outcome at end of follow-up was satisfactory in all eleven patients. In the absence of any other differences between our method and the standard technique, we believe that the use of a hollow trephine instead of the solid reamer was responsible for the reduced incidence of adhesions.

**Fig. 5:**
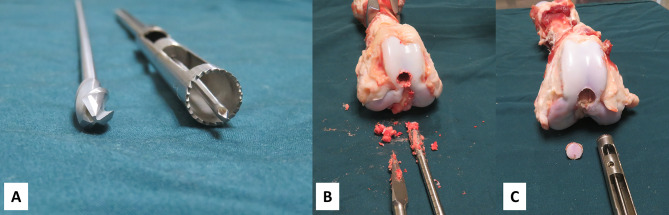
Comparison of the amount of bone debris produced by the ordinary solid reamer and a hollow trephine during hole opening in the femur of a pig A: Two types reamers: ordinary solid reamer and hollow trephine B: Many bone debris produced by solid reamer opening C: The hollow trephine produces only little bone debris and a smooth bolt during opening

The hollow trephine has several benefits. It produces less bone debris than the traditional solid reamer. Bone debris is difficult to remove, and residual bone debris can lead knee joint adhesions and heterotopic ossification [13]. According to Furlong et al., reaming debris is an important cause of heterotopic ossification after femoral intramedullary nailing [14]. Further, Kantak et al. demonstrated that removal of bone debris by extensive irrigation with normal saline can reduce the incidence of heterotopic ossification [2]. Figure 5 shows many bone debris produced by the solid reamer opening and scatters it in the knee joint cavity. However, the hollow trephine produces little bone debris and a smooth bolt during opening (Fig. 5). Although bone debris is only one of many pathogenic factors, we believe that the use of the hollow trephine can help to reduce the incidence of postoperative knee adhesion and heterotopic ossification.

Another advantage of the hollow trephine is that it can be used for bone graft harvesting. Autologous bone graft is the best choice for limb reconstruction or fusion operation. Although the iliac crest is the most common site for harvesting bone graft material, it can only provide a limited amount of cancellous bone. Harvesting large amounts from the iliac crest can lead to severe pain and infection [15]. The femur is also a good source of bone graft. The reamer-irrigator-aspirator (RIA) technique has been proposed as a method to obtain large bone grafts from the femur [16], and some orthopedists have successfully applied the technique in clinical practice [12, 17]. Liu et al. have reported the application of the hollow trephine for harvesting bone graft during the antegrade femoral nail procedure [5]. Several authors have also reported the use of RIA for retrograde femoral bone graft acquisition [18, 19]. Cepni et al. have used a manual trepan for reaming, which is a laborious process. Moreover, the lack of guide pin and depth-limiting device leads to the possibility of deflection of the opening direction [6]. The new trephine we designed is more accurate and not too deep under the guidance of the guide pin. Our in-house designed hollow trephines generate little debris in the joint cavity, but it provides a bone bolt that can be used to fill diaphyseal gaps and promote bone healing. Bone fragments produced during reaming have a similar transcriptional profile as bone grafts harvested from the ilium and these genes play important roles in bone repair [20]. If necessary, further intramedullary cancellous bone can be harvested using other tools when using retrograde intramedullary nails for revision surgery for nonunion/defect of femoral fractures. Tunnel preparation and harvesting could be finished during the reaming operation using the hollow trephine. In contrast, the scattered bone debris produced using the ordinary reamer opening is not only difficult to harvest and it also cannot be used for structural rebuilding. Bone bolt is a good source of bone graft, but all the cases in our study were closed reduction and bone graft was not used.

**Fig. 6:**
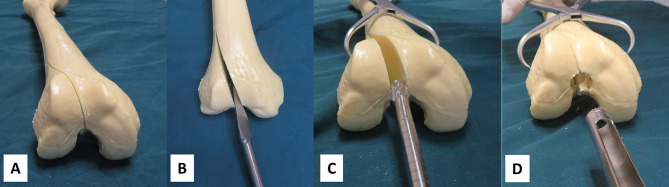
The use of a hollow trephine can avoid wedge effect A: A simple femoral intercondylar fracture (AO/OTA classification 33.B1) B: A triangular pyramid distracts fracture gap C: The reamer wedges the fracture gap D: With the same start point, the hollow trephine does not distract the fracture gap and avoids the “wedge effect”

The hollow trephine also prevents the wedge effect. The wedge effect refers to femoral shaft lateralization and varus malalignment of the neck that occurs during insertion of the solid reamer in the proximal femur [11, 21]. Eceviz et al. called it the V-effect [22]. Various measures have been proposed for addressing the problem of the wedge effect, including percutaneously threaded steel wire clamping, clamp fixation, and the use of a trephine [11, 23]. The wedge effect can be observed when a solid reamer is used to create the opening during retrograde intramedullary nailing to fix a simple femoral intercondylar fracture (AO/OTA classification 33.B1). The standard conical reamer dissipates the stress at fracture site and do not remove enough bone from the entry point. The reamer/nail wedges fracture fragment apart, leading to malreduction of the articular surface. In contrast, the hollow trephine does not distract fracture site (Fig. 6). It prevents the wedge effect by removing a bone plug from the entry site. A standard channel allows for passage of the nail without any distracting force on the distal femur.

## Limitation

This study mainly summarizes the application of the new technique in a small sample of patients, without focusing too much on functional scores and prognosis. The comparative study on the effect of a hollow trephine and the solid reamer on knee function score after intramedullary nailing will be further summarized in the future.

## Conclusions

The use of a hollow trephine instead of the solid reamer during the operation of retrograde intramedullary nailing of the femur can reduce bone debris, harvest bone graft, and avoid the wedge effect.

## Data Availability

All data generated or analysed during this study are included in this published article.

## Declarations

### A statement to confirm

All methods were carried out in accordance with relevant guidelines and regulations.
